# Withaferin-A Reduces Type I Collagen Expression In Vitro and Inhibits Development of Myocardial Fibrosis In Vivo

**DOI:** 10.1371/journal.pone.0042989

**Published:** 2012-08-10

**Authors:** Azariyas A. Challa, Milica Vukmirovic, John Blackmon, Branko Stefanovic

**Affiliations:** Department of Biomedical Sciences, College of Medicine, Florida State University, Tallahassee, Florida, United States of America; Harvard Medical School, United States of America

## Abstract

Type I collagen is the most abundant protein in the human body. Its excessive synthesis results in fibrosis of various organs. Fibrosis is a major medical problem without an existing cure. Excessive synthesis of type I collagen in fibrosis is primarily due to stabilization of collagen mRNAs. We recently reported that intermediate filaments composed of vimentin regulate collagen synthesis by stabilizing collagen mRNAs. Vimentin is a primary target of Withaferin-A (WF-A). Therefore, we hypothesized that WF-A may reduce type I collagen production by disrupting vimentin filaments and decreasing the stability of collagen mRNAs. This study is to determine if WF-A exhibits anti-fibrotic properties *in vitro* and *in vivo* and to elucidate the molecular mechanisms of its action. In lung, skin and heart fibroblasts WF-A disrupted vimentin filaments at concentrations of 0.5–1.5 µM and reduced 3 fold the half-lives of collagen α1(I) and α2(I) mRNAs and protein expression. In addition, WF-A inhibited TGF-β1 induced phosphorylation of TGF-β1 receptor I, Smad3 phosphorylation and transcription of collagen genes. WF-A also inhibited *in vitro* activation of primary hepatic stellate cells and decreased their type I collagen expression. In mice, administration of 4 mg/kg WF-A daily for 2 weeks reduced isoproterenol-induced myocardial fibrosis by 50%. Our findings provide strong evidence that Withaferin-A could act as an anti-fibrotic compound against fibroproliferative diseases, including, but not limited to, cardiac interstitial fibrosis.

## Introduction

Fibroproliferative disorders are major causes of morbidity and mortality globally [1], [2]. Fibroproliferative disorders affect all tissues and organ systems, include liver cirrhosis, interstitial lung diseases, chronic renal diseases, and several cardiovascular diseases [3], [4], [5]. In addition to their high prevalence, fibrotic diseases typically have severe and progressive nature [6]. Despite the huge impact of these diseases on human health, there are currently no anti-fibrotic therapies approved for use in humans [7].

Excessive collagen deposition is the hall mark of all fibroproliferative disorders [8]. Activated fibroblasts and myofibroblasts are the most important cells depositing type I collagen in all tissues. Increased activity of profibrotic cytokines such as TGF-β1 and IL-13, are implicated in the activation and differentiation of fibroblasts in to myofibroblasts, as well as in mediating the upregulation of type I collagen in these cells [9]. Increased expression of type I collagen from activated fibroblasts and myofibroblasts is regulated both, at the level of transcription and post-transcriptionally [10]. Transcription of collagen genes increases 3–10 fold in activated fibroblasts [11]. The increase in the stability of collagen mRNAs during activation contributes even more to the high expression. For instance, the dramatic increase in steady state level of collagen mRNAs during activation of hepatic stellate cells (HSC, also known as Ito or fat-storing cells) is mainly attributed to prolongation of the half-lives of collagen mRNAs from 1.5 h to greater than 24 h [12], [13]. The increased production of collagen by skin fibroblasts from scleroderma patients is also, primarily, due to an increase in stability of type I collagen mRNAs [14].

The stem-loop of the 5′ untranslated region (UTR) of collagen α1(I) and α2(I) mRNAs (5′SL) is the key element regulating their stability and translation. LA Ribonucleoprotein domain family member 6 (LARP6), binds the 5′SL of collagen mRNAs with high affinity and specificity [15]. We recently identified vimentin as key molecule involved in posttranscriptional regulation of collagen expression [16]. We showed that vimentin filaments bind collagen mRNAs in a LARP6 dependent manner and that the integrity of these filaments is crucial for stability of type I collagen mRNAs. The knockout of vimentin in mouse embryonic fibroblasts led to significantly decreased collagen I production, due to the decreased half-life of collagen I mRNAs. Likewise, disrupting vimentin filaments by overexpression of dominant negative desmin protein or by treatment of cells with β,β′-iminodipropionitrile led to a marked reduction in collagen synthesis. Based on these results, we suggested targeting vimentin filaments can be an effective anti-fibrotic therapy.

Withaferin-A (WF-A) is a steroidal lactone and the principal active ingredient of the herbal plant, Withania sominifera [17]. The intermediate filament vimentin is the primary target of WF-A. WF-A binds vimentin and covalently modifies a conserved cysteine residue located in the α-helical rod 2B domain of vimentin [18]. Further, WF-A treatment disrupts a vimentin filament network in endothelial cells. In light of our finding of a role of vimentin in stabilizing collagen mRNAs, we hypothesized that WF-A may reduce collagen production by disrupting vimentin filaments and decreasing the stability of collagen mRNAs [16]. No previous study investigated the effect of WF-A on collagen synthesis and/or fibrosis.

The aim of the present study was to determine if W-A exhibits anti-fibrotic properties *in vitro* and *in vivo* and to elucidate the molecular mechanisms by which WF-A exerts its anti-fibrotic effects. We report that, in tissue culture, WF-A suppresses collagen I expression, both at transcriptional and post-transcriptional level, by inhibiting the TGF-β signaling pathway and by disrupting vimentin filaments, respectively. Importantly, *in vivo*, we demonstrate that WF-A attenuates cardiac fibrosis in a mouse model of isoproterenol induced myocardial fibrosis.

## Materials and Methods

### Cells

Primary human lung fibroblasts (HLF) immortalized by expression of telomerase reverse transcriptase [19] were grown under standard conditions. Scleroderma fibroblasts were derived from skin of a scleroderma patient and were purchased from the European collection of cell cultures (cell line BM0070). Mouse embryonic fibroblasts from wild type (VIM+/+MEFs) and vimentin knock-out mice (VIM−/−MEFs) were described before [20]. They were derived from day 12–13 vimentin −/− and vimentin +/+ mouse embryos, after transfection with an expression vector encoding SV-40 early genes [20]. One cell line isolated from the vim+/+ fibroblasts and one cell line isolated from the vim−/− fibroblasts were kind gifts of Dr. Robert Evans, University of Colorado, and were used in this study. Cardiac fibroblasts were isolated from 200–250 g female Sprague–Dawley rats as described [21] and passaged 2–3 times before use in the experiments. All cells were cultured in Dulbecco’s Modified Eagle’s Medium, supplemented with 10% fetal bovine serum. Cultured cells were treated with DMSO or different concentrations of WF-A (0.25–3.0 µM) for 24 h. For TGF-β stimulation, HLFs were cultured in a low serum medium (1% FBS) for 24 h. Cells were treated with DMSO or WF-A (1.0–1.5 µM) 1 h before adding TGF-β1 (R&D Systems, Minneapolis, MN) at a concentration of 5 ng/ml and the incubation was continued for additional 18–24 h. Rat HSC were isolated by perfusion of the liver with collagenase and pronase, followed by centrifugation over a Nycodenz gradient, as described [22]. Isolated HSC were cultured in DMEM supplemented with 10% FBS for two days when WF-A was added to medium. Cells were harvested after additional 4 days, with total of 6 days in culture.

### Chemicals

Withaferin-A (Chromadex, Santa Ana, CA) was dissolved in DMSO at 3 µM and stored at −20°C. For the *in vivo* experiments, WF-A was dissolved in a vehicle containing 10% ethanol, 40% DMSO and 50% Cremephor as previously described [23]. Isoproterenol (Sigma-Aldrich) was dissolved in 0.9% saline for use in the *in vivo* experiments. SB203580, a specific p38 MAPK inhibitor [24], was obtained from Sigma and was used at a concentration of 10 µM.

### Cell Viability Assay

Apoptosis of fibroblasts following WF-A treatment was assessed by measuring Caspase 3 and Caspase 7 activities using a Caspase-Glo 3/7 assay kit (Promega, Madison, WI) according to the manufacturer’s procedures. Briefly, 2×10^4^ cells/well were plated into white-walled 96-well plates and incubated for 24 h under normal growth conditions. Subsequently, cells were treated with DMSO or various concentrations of WF-A (0.25–3.0 µM), and the plates were incubated for additional 24 h. After the 24 h incubation, the Caspase-Glo 3/7 reagent was added to the wells, and luminescence was measured with a plate-reading luminometer after 1 h incubation. Three separate experiments done in duplicates were performed and the data were expressed as relative light units and shown as the percentage of light units of untreated cells.

### Luciferase Assay

One TGF-β inducible Luciferase reporter construct contained the 378 bp of the α2(I) collagen (COL1A2) promoter and 58 bp of the transcribed sequence fused to the luciferase (Luc) open reading frame. The mutant COL1A2-LUC harbored a mutation in the Smad binding site of the TGF-beta response element within this promoter. These constructs did not contain the 5′ stem-loop. Both plasmids were kind gifts from Dr. Francesco Ramirez, Mount Sinai School of medicine and were described before [25]. Another TGF-β responsive plasmid ((CAGA)_12_MLP-Luc) contained 12 CAGA boxes cloned upstream of the initiator sequence of the adenovirus major late promoter (MLP) and was a kind gift of Dr. Peter ten Dijnke. This construct was also described before [26]. Primary HLFs were seeded at a density of 10,000 cells/cm^2^ and cultured in 1% FBS. Cells were co-transfected using Lipofectamine 2000 (Invitrogen) with 0.2 µg of β-galactosidase plasmid and 0.8 µg of the Luciferase reporter plasmids described above. TGF-β1 (5 ng/ml) was added 24 h after transfections and luciferase readings were taken 16–24 h after that. To standardize the results for transfection efficiency, β-galactosidase activity was measured according to the standard protocol [27] and luciferase activity was normalized to the β-gal activity.

### TGF-β Bioassay

Mink Lung Epithelial Cells (MLECs) stably transfected with TGF-β1 responsive luciferase reporter gene driven by plasminogen activator inhibitor-1 promoter were kind gifts of Dr. Daniel Rifkin, NYU. The use of these cells for measuring TGF-β1 activity has been described before [28]. Mature TGF-beta binds to the receptors of MLEC transfected with a plasminogen activator inhibitor-1 promoter-luciferase construct (PAI/L), resulting in a dose-dependent increase of luciferase activity [28]. Briefly, MLECs were seeded at 60,000 cells/cm^2^ and serum starved for 24 hrs. The cells were then incubated with conditioned medium collected from cultured HLFs, which had been treated with varying concentrations of WF-A. TGF-β1 activity was assessed in cell lysates 16 h later by measuring the luciferase activity. MLECs were incubated with unconditioned medium or MLECs were treated with increasing concentrations of pure TGF-β1 and were measured as negative and positive controls, respectively. The results were performed in triplicates.

### Western Blot Analysis

Cells were lysed using RIPA buffer containing 50 mM Tris PH 7.4, 150 mM NaCl, 0.5% sodium deoxycholate, 1% NP-40, 0.1% SDS, 1 mM EDTA, and mix of protease inhibitors. Protein concentration was estimated by the Bradford assay, with bovine serum albumin as the standard. A 40 to 50 µg of total cellular protein was typically used for Western blot analysis. Anti-Smad3 and anti-phospho-Smad3 Ser423/425 monoclonal antibodies were obtained from Cell Signaling. Anti-collagen α1(I) antibody was obtained from Rockland; anti-collagen α2(I) antibody specific for human polypeptide was from Cell Signaling; the anti-collagen α2(I) and the anti-collagen α1(III) antibodies to detect mouse polypeptides were obtained from Santa-Cruz Biotechnology. Anti-vimentin antibody, anti-smooth muscle actin and anti-actin antibodies were obtained from Abnova. For determination of phosphor-Smad3, cells were lysed with lysis solution containing phosphatase inhibitors (NaF and Na3VO4). For Western blots with tissue samples, TRI-reagent (Sigma) was used to lyse and extract proteins from the snap-frozen heart tissue specimens. Densitiometric analysis of scanned images from films (Western blots) was performed by Image J software.

### Reverse Transcription-PCR Analysis

Total cellular RNA was isolated using an RNA isolation kit (Sigma). For extraction of RNA from heart tissues, the extraction was done using Tri-reagent (Sigma). RT-PCRs were performed with 100 ng of total RNA and using rTth reverse transcriptase (Boca Scientific, Boca Raton, Fl). [^32^P]dCTP was included in the PCR step to label the products, which were resolved on sequencing gels, as described previously [29]. The number of cycles was adjusted within the linear range of the reaction. Reactions run in 6% urea gel were exposed for autoradiography and quantified using typhoon phosphorimager. The primers used for RT-PCR are shown in a Table 1.

**Table 1:** Primers used for RT-PCR and QRT-PCR.

*h-collagen á1(I)*	F: AGAGGCGAAGGCAACAGTCG and, R: GCAGGGCCAATGTCTAGTCC
*h-collagen á2(I)*	F: CTTCGTGCCTAGCAACATGC and, R: TCAACACCATCTCTGCCTCG
*h-collagen á1(III)*	F: ATCTTGGTCAGTCCTATGCGG and, R: GCAGTCTAATTCTTGATCGTCA
*h-actin*	F: GTGCGTGACATTAAGGAGAAG and, R: GAAGGTAGTTTCGTGGATGCC
*h-fibronectin*	F: ACCAACCTACGGATGACTCG and, R: GCTCATCATCTGGCCATTTT
*h-TGFâ-1*	F: GGGACTATCATCCACCTGCAAGA and, R: CCTCCTTGGCGTAGTAGTCG
*h-TGFâR1*	F: GGGGAAACAATACTGGCTGA and, R: GAGCTCTTGAGGTCCCTGTG
*h-TGFâRII*	F: GCAAGTTTTGCGATGTGAGA and, R: GGCATCTTCCAGAGTGAAGC
*m-collagen á1(I)*	F: GAGCGGAGAGTACTGGATCG and, R: TACTCGAACGGGAATCCATC
*m-collagen á2(I)*	F: CTTCGTGCCTAGCAACATGC and, R: TCAACACCATCTCTGCCTCG
*m-collagen á1(III)*	F: ACGTAAGCACTGGTGGACAG and, R: AGCTGCACATCAACGACATC
*m-fibronectin*	F: AATGGAAAAGGGGAATGGAC and, R: CTCGGTTGTCCTTCTTGCTC
*m-actin*	F:CGTGCGTGACATCAAAGAGAAGCand,R:TGGATGCCACAGGATTCCATACC
*m-á-SMA*	F: CTGACAGAGGCACCACTGAA and, R: CATCTCCAGAGTCCAGCACA
*m-TGFâ-1*	F: TTGCTTCAGCTCCACAGAGA and, R: TGGTTGTAGTGGGCAAGGAC
*m-TGFâRI*	F: GGCGAAGGCATTACAGTCTT and, R: TGCACATACAAATCGCCTGT
*m-TGFâRII*	F: GCAAGTTTTGCGATGTGAGA and, R: GGCATCTTCCAGAGTGAAGC
*m-smad2*	F: GGAACCTGCATTCTGGTGTT and, R: ACGTTGGAGAGCAAGCCTAA
*r-collagen á1(I)*	F: TGAGCCAGCAGATTGAGAAC and, R: TGATGGCATCCAGGTTGCAG
*r-collagen á2(I)*	F: CTCACTCCTGAAGGCTCTAG and, R: CTCCTAACCAGACATGCTTG
*r-actin*	F:CGTGCGTGACATTAAAGAGAAGCand,R:TGCATGCCACAGGATTCCATACC
*r-GAPDH*	F: ACCGGTTCCAGTAGGTACTG and, R: CTCACCGTCACTACCGTACC
*r-á-SMA*	F: ACAGAGAGAAGATGACGCAG and, R: GGAAGATGATGCAGCAGTAG

F, forward primer; R, reverse primer; h, human; m, mouse; r, rat; and SMA, smooth muscle actin.

### Quantitative Real-time qRT-PCR Analysis

Total cellular RNA was isolated using an RNA isolation kit (Sigma). RNA was treated with DNAse I to remove residues of DNA upon RNA isolation and equal amounts of RNA were used in reverse-transcription reaction. The cDNA was synthetized using SuperScript II RT (Invitrogen) following the manufacturer’s protocol. Five percent of total CDNA was used in quantitative polymerase chain reaction (qRT-PCR) in BioRad–IQ5 Thermocycler. The sequences of the primers used in qRT-PCR reaction are presented in Table 1. The qRT-PCR reactions were performed in duplicates. The threshold cycle (Ct) was calculated using IQ-5 software (Bio-Rad) with standard curves constructed for each primer set with a stepwise dilution of input DNA. The samples were subjected to 40 cycles of qRT-PCR. The total collagen α1(I) and α2(I) mRNA levels were standardized to the actin level and the negative control (empty vector). The equation for determination of standard error of the mean (SEM) for normalized values and graphics were computed using biostatic program GraphPad Prism 3.02. One way ANOVA method, followed by Turkey’s multiple comparison test, p<0.05, was used to determine significant differences. The P-values were determined by the Student’s t-test.

### Determination of RNA Stability

Cells were treated with Actinomycin D (Act-D) (10 µg/ml) for 0, 6, 12 and 24 h. After the Act-D treatment, the cells were scraped; a total RNA was extracted and analyzed by RT-PCR. RNA extracted from cells at time point 0 (immediately after the addition of Act-D) was used as the initial level of mRNA and arbitrarily set as 100%. Results from three independent experiments are used to plot the histograms.

### Immunostaining

Cells were seeded onto glass coverslips. After treatment, the cells were fixed with 4% formaldehyde for 30 min at room temperature and permeabilized with 0.5% Triton X-100 in phosphate buffered saline (PBS) for 10 min. Blocking was done with 10% goat serum/5% bovine serum albumin in PBS for 1 h at room temperature, followed by overnight incubation with anti-vimentin antibody at 4°C. After washing, cells were incubated with AlexaFluor 594-conjugated secondary antibody diluted in a blocking solution. Cells were, then, mounted using Prolong mounting solution containing 4′, 6′-diamidino-2-phenylindole (DAPI) (Invitrogen). Images were taken by the Leica TCS SP2 AOBS laser confocal microscope equipped with a Chameleon Ti-Sapphire multiphoton laser. Optical sections were processed with LCSLite software and single plane confocal images are shown.

### Animal Studies

Twelve weeks old (25–30 grams) 129 Svev male mice (n = 24) were obtained from the Charles River (MA, US). The mice were housed in a standard condition with 12 h dark-light cycle and were given standard diet with ad libitum access to food and water. The mice were randomly divided into four groups; Isoproterenol group (ISO) (n = 6), Isoproterenol and WF-A group (ISO+WF-A) (n = 6), WF-A only group (WF-A) (n = 6) and vehicle group (CONT) (n = 6). The protocol for this study was approved by the Florida State University Animal Care and Use Committee; protocol number 1025. All animal procedures and experiments were performed under the NIH guidelines for animal care and use. Isoproterenol (50 mg/kg) was injected subcutaneously to the ISO and the ISO+WF-A groups for 2 consecutive days, whereas mice in the WF-A and CONT groups received subcutaneous 0.9% saline injections. WF-A (4 mg/kg) was injected intraperitoneally to the ISO+WF-A group and to the WF-A group daily for 14 days, whereas vehicle IP injections were given to the mice in the ISO and CONT groups. Twenty four hours after the last injection, mice were sacrificed by an overdose of pentobarbital (50 mg/kg ip). The heart was removed and weighed. The weight was used to determine heart weight to body weight (HW/BW) ratio. The atria were removed and a transverse section at the level of the papillary muscles was snap-frozen in liquid nitrogen for Western blot and RT-PCR analysis. The remaining ventricle including the apex was fixed minimum of 24 h with 10% buffered formalin for histopathology analysis.

### Histopathology Analysis

Fixed heart tissue was embedded in paraffin and transverse sections (5 µm) from the apex of the ventricle as well as from the middle zone of the ventricle were placed on microscope slides. Slides were processed for Masson’s trichrome or H&E staining. To determine the degree of cardiac fibrosis, 20X magnification images were analyzed using Image J software with Threshold-color plug-in. The area of blue stained region (fibrosis) was divided by the total area to obtain percentage of cardiac fibrosis.

### Statistical Analysis

Data are presented as mean ± SEM. For the *in vitro* experiments, results from a minimum of three independent experiments were analyzed. Differences between two groups were analyzed for statistical significance using the Student’s t test. Comparisons among groups (i.e. more than two groups) were made by ANOVA method. Statistical values of p<0.05 were considered to be significant.

## Results

### Withaferin-A Disrupts Vimentin Filaments and causes Degradation of Vimentin in Fibroblasts

It has been reported that WF-A causes disruption of the vimentin network in different cell types including endothelial cells, astrocytes and vimentin positive breast cancer cell lines [18], [30], [31]. However, the effect of WF-A on the integrity of vimentin network in fibroblasts has not been studied. We, thus, determined the effect of WF-A on integrity of vimentin filaments in fibroblasts.

**Figure 1:**
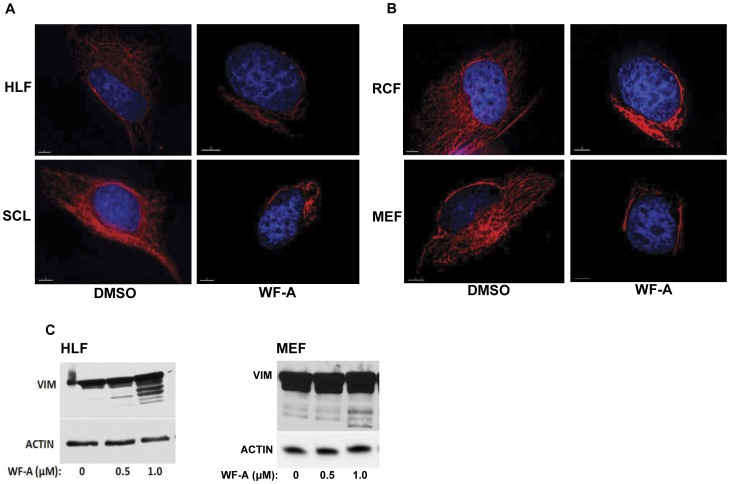
Effect of WF-A on integrity of vimentin filaments in fibroblasts. A. Disruption of vimentin filaments in human fibroblasts treated with WF-A. Primary human lung fibroblasts (HLF, upper panels) and scleroderma fibroblasts (SCL, lower panels) were treated for 2 h with DMSO or with 1.0 µM of WF-A and immunostained with anti-vimentin antibody. Nuclear staining was by DAPI (blue). Bars, 1 µm. B. Collapse of vimentin filaments in rodent fibroblasts treated with WF-A. Experiment as in A, except primary rat cardiac fibroblasts (RCF, upper panels) and mouse embryonic fibroblasts (MEF, lower panels) were treated with DMSO or 1.0 µM of WF-A. Bars, 1 µm. C. Degradation of soluble vimentin by WF-A. Soluble vimentin was extracted with high salt buffer from HLF (left) and MEF (right) after treatment with the indicated concentrations of WF-A for 2 h. Vimentin (VIM) was analyzed by Western blot. Loading control: actin.

Treatment of human lung fibroblasts (HLFs) with 1.0 µM Withaferin-A caused complete collapse of the vimentin network, observed 2 h after treatment (Figure 1A, upper image). Similar results were obtained in scleroderma fibroblasts (SCL) (Figure 1A, lower images), rat cardiac fibroblasts (RCF) (Figure 1B, upper images) and mouse embryonic fibroblasts (MEFs) (Figure 1B, lower images) suggesting that WF-A can disrupt vimentin filaments in fibroblasts of different sources. Disruption of vimentin network by WF-A induced a change in cell shape of the fibroblasts. WF-A treated fibroblasts had a flattened and elongated morphology in comparison to non-treated fibroblasts (Figure S1A and S1B).

In addition to disrupting vimentin filaments, in endothelial and glial cells, WF-A caused degradation of soluble vimentin [18], [30]. We tested if WF-A can cause degradation of soluble vimentin in HLFs and MEFs. Vimentin was separated into soluble and insoluble fractions and the soluble fraction was analyzed by Western blot [16]. The total amount of soluble vimentin wasn’t decreased possibly due to contamination from the more abundant insoluble vimentin. However, WF-A caused appearance of lower molecular products of soluble vimentin in a dose-dependent manner, suggesting that WF-A can cause degradation of vimentin in HLFs and MEFs (Figure 1C).

### Study of the Toxicity of Withaferin-A in Cultured Fibroblasts

**Figure 2:**
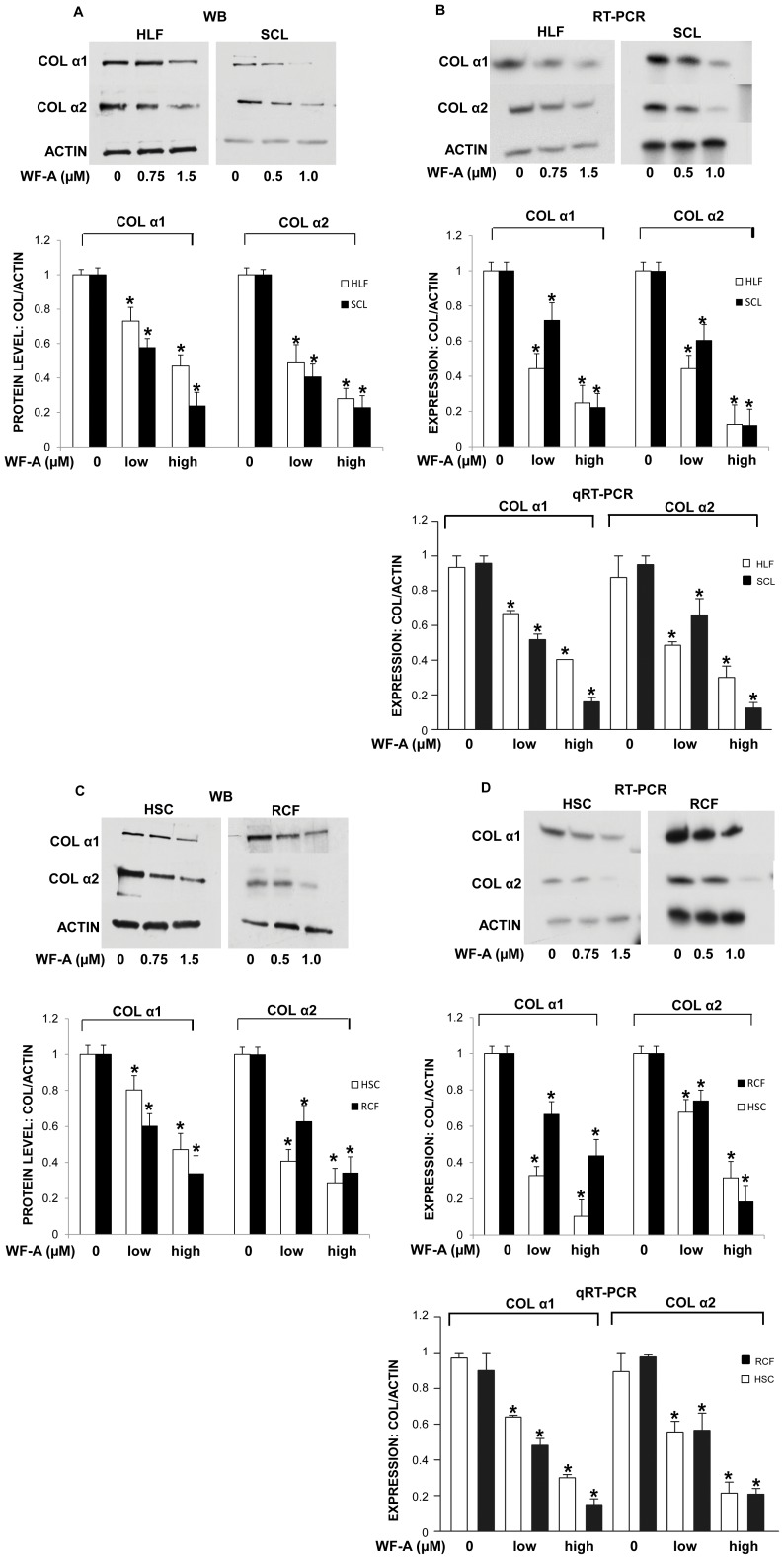
WF-A reduces expression of type I collagen α1 and α2 polypeptides and their mRNAs in human and rodent fibroblasts. A. WF-A reduces collagen type I protein in human fibroblasts. HLF (upper left panel) and SCL fibroblasts (upper right panel) were treated WF-A and cellular levels of collagen α1(I) (COLα1) and α2(I) (COLα1) polypeptides were analyzed by Western blotting. Loading control: actin. Bottom panels; the expression of collagen polypeptides were normalized to actin and plotted from three independent experiments. Open bars: HLF, black bars: SCL. Low concentrations of WF-A: (0.75 µM for HLF) and (0.5 µM for SCL). High concentrations of WF-A: (1.5 µM for HLF) and (1.0 µM for SCL). The error bars: ±1 SEM are shown. B. WF-A decreases the steady-state levels of collagen α1 and α2 mRNAs in human fibroblasts. Experiment as in A, mRNAs were analyzed by RT-PCR (upper panels). Middle panel is quantification of RT-PCR from three independent experiments, and the bottom panel show quantification by real time (qRT-PCR). C. WF-A reduces collagen protein in primary rat hepatic stellate cells (HSC) and primary rat cardiac fibroblasts (RCF). HSC and RCF were isolated from rat liver and heart, respectively, and treated with the indicated amounts of WF-A. Experiment as in A. Loading control: actin. Bottom panels: levels of collagen α1(I) (COLα1) and collagen α2(I) (COLα2) polypeptides were normalized to actin and plotted from three independent experiments. Open bars: HSC, black bars: RCF. Low concentrations of WF-A: (0.75 µM for HSC) and (0.5 µM for RCF). High concentrations: (1.5 µM for HSC) and (1.0 µM for RCF). The error bars: ±1 SEM are shown. D. WF-A decreases the steady-state levels of collagen mRNAs in HSC and RCF. Experiment as in C, mRNAs were analyzed by RT-PCR (upper panels, with quantification from three experiments shown in middle panel) and qRT-PCR (bottom panel), * represents p<0.05.

Withaferin-A has previously been reported to cause apoptosis in various normal and cancer cell types, particularly at higher concentrations [31], [32]. Therefore, it is important to determine the concentrations of WF-A that induce apoptosis in human and rodent collagen producing cells, to avoid artifacts due to cell cytotoxicty. Cells were cultured in the presence of various concentrations of WF-A (0.25–3.0 µM) for 24 h and viability of cells was determined using the caspase glo 3/7 assay kit. Viability of primary human lung fibroblasts (HLF) 24 h after treatment with the indicated concentrations of Withaferin-A is shown in the Figure S2. Withaferin-A in concentrations less than 1.5 µM did not cause significant cell death of HLFs (Figure S2A). Concentrations above 1.5 µM, however, caused increased loss of cells due to apoptosis in dose dependent manner. At 2.0 µM concentration of Withaferin-A, approximately 10% of HLFs showed apoptosis, whereas the 3.0 µM concentration of WF-A caused 15% apoptosis (Figure 2A). Similar results were obtained when rat hepatic stellate cells (HSC) were treated with WF-A (Figure S2E). These results were consistent with the result reported for human dermal fibroblasts, where only doses greater than 1.5 µM were found to be toxic [23]. In Scleroderma fibroblasts, the sub-toxic concentration was less than 1.0 µM (Figure S2B). WF-A at concentrations below 1.5 µM did not significantly affect the viability of primary rat cardiac fibroblasts (Figure S2C). We also tested the toxicity of WF-A in wild type (VIM+/+) and vimentin deficient (VIM−/−) mouse embryonic fibroblasts (MEFs). Concentrations of WF-A greater than 1.0 µM were found to be toxic in VIM+/+ as well as VIM−/− MEFs (Figure S2F). These results indicate that concentrations of WF-A of 1.0 µM or less are sub-toxic for all cell types tested, but were sufficient to collapse the vimentin network (Figure 1A and 1B).

### Withaferin-A Significantly Reduces Expression of Type I Collagen in Fibroblasts from Different Tissues

To evaluate the effect of WF-A on collagen expression, we used only the sub-toxic concentrations. We first tested the effect of WF-A on collagen expression in cultured primary human lung fibroblasts (HLF) and primary fibroblasts from skin of a scleroderma patient (SCL). These cells were treated with WF-A for 24 h (Figure 2A) and cell extracts were analyzed for collagen α1(I) and α2(I) polypeptides by Western blot. WF-A dose dependently reduced production of both, α1(I) and α2(I) polypeptides from HLFs (Figure 2A, upper left panel) as well as from SCL fibroblasts (Figure 2A, upper right panel). Actin levels were not affected and are shown as loading control. Densitometric analysis of the bands is shown in the lower panel of Figure 2A. At 1.5 µM, WF-A reduced expression of collagen α1(I) and α2(I) collagen polypeptides 2–3 fold in HLF, while in SCL fibroblasts 1 µM of WF-A reduced collagen expression 4 fold.

Total RNA from the HLFs and SCL cells was also analyzed for the steady state levels of collagen mRNAs by RT-PCR. Congruent with the effect on the polypeptides, WF-A decreased the steady state levels of collagen α1(I) and α2(I) mRNAs 3–4 fold in a dose-dependent manner in both HLFs (Figure 2B, upper left panel) and SCL fibroblasts (Figure 2B, upper right panel). The steady state level of the control β-actin mRNA was unaltered. Densitometric analysis of the bands is shown in the middle panel. To analyze the samples more quantitatively we also performed real time RT-PCR analysis. Real time RT-PCR showed similar results, collagen mRNAs were decreased 2–4 fold (Figure 2B, lower panel).

To confirm the above results in cells of different tissue of origin, we prepared activated hepatic stellate cells (HSC) from rat livers [22] and cardiac fibroblasts (RCF) from rat hearts [21]. Activated HSC were treated with WF-A at concentrations up to 1 µM for 24 h and whole cell extract and total RNA were analyzed for expression type I collagen. WF-A caused 3–4 fold reduction in the expression both collagen polypeptides in HSC (Figure 2C, upper left panel).

Cardiac fibroblasts were isolated from rat hearts [21] and at the 3rd passage the cells were treated for 24 h with WF-A at concentration of 0–1.5 µM. The treatment of primary RCF with WF-A caused 3–4 fold reduction in the expression of type I collagen polypeptides (Figure 2C, upper right panel). Densitometry of the bands is shown in the lower panel of Figure 2C.

Expression of collagen α1(I) and α2(I) mRNA was also analyzed by RT-PCR (Figure 2D, upper panel). Densitometry of the bands indicated that collagen mRNAs were reduced 2–4 fold (Figure 2D, middle panel). The analysis was repeated using real time RT-PCR. Real time RT-PCR verified the result, showing that collagen mRNAs were reduced 2–5 fold (Figure 2D, lower panel).

From the results using different primary collagen producing cells, we concluded that WF-A can potently inhibit the expression of type I collagen regardless of the tissue of origin.

### Withaferin-A Increases the Rate of Decay of α1(I) and α2(I) Collagen mRNAs

**Figure 3:**
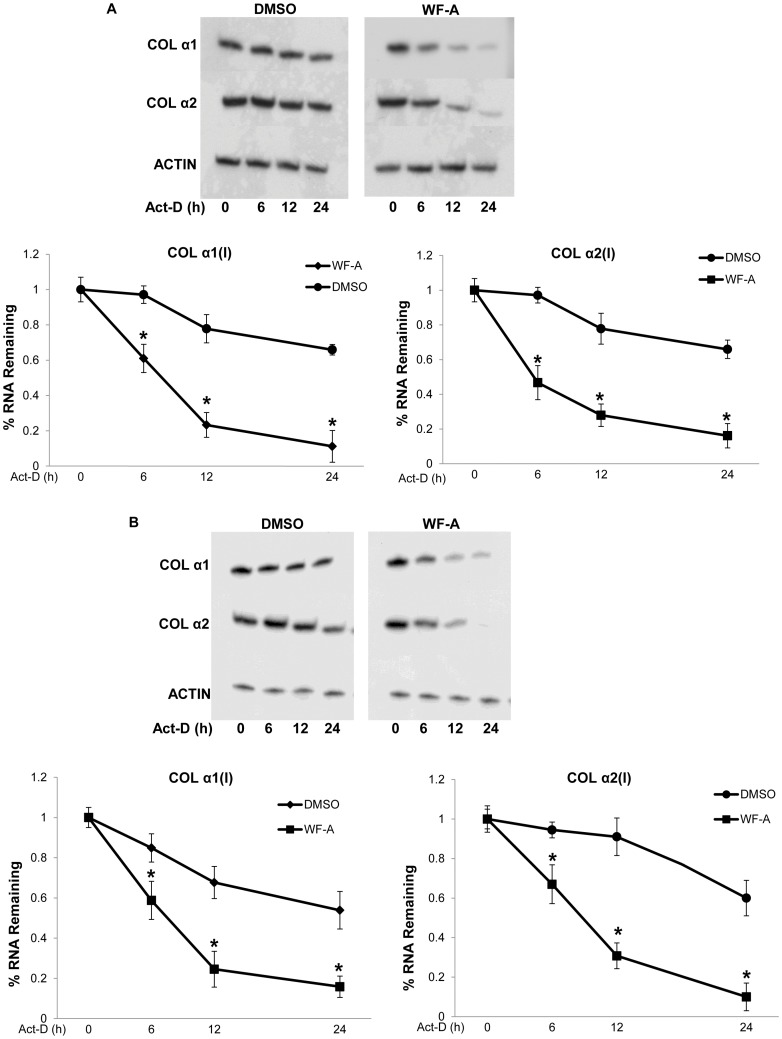
WF-A destabilizes collagen α1(I) and α2(I) mRNAs in human fibroblasts. A. Stability of type I collagen mRNAs in WF-A treated HLF. HLF treated with DMSO (upper left panel) or 1.5 µM WF-A (upper right panel) for 24 h, transcription was blocked with Act D and collagen α1(I) and α2(I) mRNAs were measured by RT-PCR at the indicated times after the transcription block. Actin mRNA was measured as a loading control. Bottom panels: decay rates of collagen α1(I) and α2(I) mRNAs were estimated in DMSO and WF-A treated cells in three independent experiments. Expression of collagen mRNAs was normalized to the expression of actin mRNA and plotted as function of time after the transcription block. The levels at time 0 were arbitrarily set as 1. Error bars represent ±1SEM. B. Stability of collagen mRNAs in WF-A treated SCL fibroblasts. The experiments as in A, except SCL fibroblasts were used. * represents p<0.05.

Since WF-A disrupted vimentin filaments, we tested if the reduced level of collagen mRNAs following treatment with WF-A is due to their increased turnover. We determined the stability of collagen mRNAs after WF-A treatment and transcription block by Actinomycin-D (Figure 3A, upper panels). The lower panels in Figure 3A show quantification of the densitometric scans of three experiments. Collagen mRNAs have half-lives between 18–27 h in normal collagen producing cells [12]. In the DMSO treated HLFs, the half-life of collagen α1(I) mRNA was estimated to be around 24 h. The half-life of collagen α2(I) mRNA was estimated to be more than 24 h, as the level dropped to about 60%, 24 h after the transcription block However, in HLFs treated with 1.5 µM WF-A, the half-life of collagen α1(I) mRNA was reduced 3-fold, to approximately 8 h, while the half-life of collagen α2(I) mRNA was reduced 4-fold, to about 6 h. Similar results were obtained in scleroderma fibroblasts where the half-lives of both collagen α1(I) and α2(I) mRNAs were reduced from 24 h to about 9 h (Figure 3B). We, therefore, demonstrated that WF-A reduces stability of collagen mRNAs in two types of fibroblasts and that the down-regulation of collagen expression by WF-A is, at least in part, mediated by its ability to reduce the half-life of collagen mRNAs. The above results are consistent with the report that cells, in which vimentin filaments are disrupted, have greatly reduced half-life of collagen mRNAs [16]. It is therefore likely that the destabilizing effect of WF-A on the collagen mRNAs is mediated by its ability to disrupt vimentin filaments.

### The Effect of Withaferin-A on Collagen mRNA Stability is Dependent on its Effect on Vimentin

**Figure 4:**
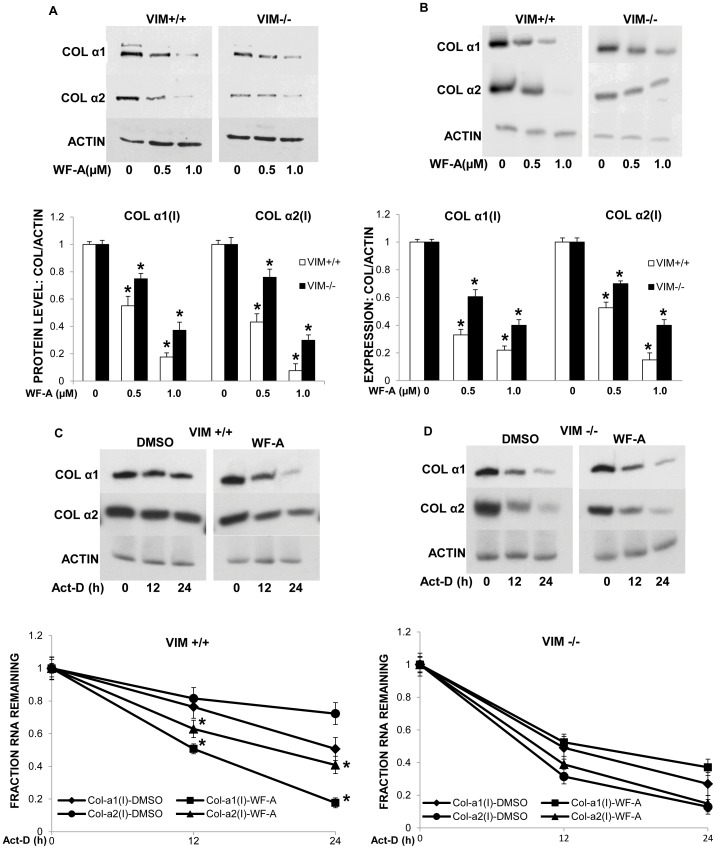
Effects of WF-A on collagen expression in vimentin knock-out fibroblasts. A. Expression of collagen polypeptides in vimentin knock-out fibroblasts treated with WF-A. Wild type mouse embryonic fibroblasts (VIM+/+ MEFs, upper right panel) and vimentin knock-out (VIM−/− MEFs, upper left panel) were treated with WF-A and collagen α1(I) and α2(I) polypeptides analyzed by Western blotting. Loading control: actin. Bottom panel: the expression of collagen α1(I) and α2(I) polypeptides from VIM+/+ MEFs (open bars) and VIM−/− MEFs (black bars) was normalized to actin expression andresults from three independent experiments were plotted. The error bars represent ±1SEM. B. Effect of WF-A on expression of collagen mRNAs in VIM+/+ and VIM−/− MEFs. Experiment as in A, except mRNAs were analyzed by RT-PCR. C. WF-A destabilizes collagen mRNAs in VIM+/+ MEFs. VIM+/+ MEFs were treated with DMSO (left panel) or 1.0 µM WF-A (right panel) for 24 h. Transcription was then blocked by Act-D and collagen α1(I) and α2(I) mRNAs were measured at the indicated times after the block by RT-PCR. Actin mRNA was measured as a loading control. Bottom panel: VIM+/+ MEFs treated with WF-A (full lines) or DMSO (broken lines) and the levels of collagen α1(I) mRNA (circles) and α2(I) mRNA (squares) were normalized to actin mRNA and plotted for the indicated time points after the transcription block. The level at time point 0 was arbitrarily set as 1. The error bars represent ±1 SEM, estimated from three independent experiments. D. WF-A has no effect on stability of collagen mRNAs in VIM−/− MEFs. Experiment as in C, except in VIM−/− MEFs were used. * represents p<0.05.

Withaferin-A may have variety of intracellular effects not related to disrupting vimentin filaments as suggested by its ability to induce apoptosis at higher concentrations. Although our results clearly show that WF-A reduced the stability of collagen mRNA, it is possible that the effect of WF-A on half-life of collagen mRNAs may be independent of the integrity of vimentin filaments. To test if targeting vimentin is a prerequisite for effects of WF-A on stability of collagen mRNAs, we compared effect of WF-A on expression of collagen polypeptides in VIM+/+ MEFs and VIM−/− MEFs. In VIM+/+ MEFs, WF-A caused a dose dependent reduction in the levels of collagen α1(I) and α2(I) polypeptides (Figure 4A, upper left panel). Densitiometric analysis showed that at 0.5 µM WF-A, there was 50–60% reduction at the level of collagen α1(I) and α2(I) polypeptides and that at 1.0 µM of WF-A there was about 80–90% reduction (Figure 4A, lower panel). In VIM−/− MEFs, at 0.5 µM of WF-A there was 30% reduction and at 1.0 µM of WF-A, there was 60–70% reduction (Figure 4A, upper right panel and lower panel for quantification). This result suggested that WF-A reduces collagen expression in VIM+/+ cells to a greater extent than in VIM−/− cells.

We also analyzed the effect of WF-A on collagen mRNA levels in VIM+/+ and VIM−/− MEFs. Again, the level of collagen mRNAs was reduced to a greater extent in VIM+/+ MEFs then in VIM−/− MEFs (Figure 4B). Therefore, we concluded that some of the effects of WF-A on collagen expression are dependent on the presence of vimentin filaments.

Since the steady state level of collagen mRNAs reflects both transcription and stability of the mRNAs, we wanted to assess which process is responsible for the difference seen in WF-A treated VIM+/+ and VIM−/− MEFs. To this end, we compared the effect of WF-A on stability of collagen mRNAs in VIM−/− and s VIM+/+ MEFs. In VIM+/+ MEFs treated with 1.0 µM WF-A the half-life of both α1(I) and α2(I) collagen mRNAs was reduced by about 50% compared to the cells treated with DMSO. Figure 4C shows one representative experiment (upper panel) and quantification from three experiments (lower panel). However, in a similar experiment in VIM−/− cells, half-lives of both α1(I) and α2(I) collagen mRNAs were shorten in comparison to half-lives of α1(I) and α2(I) collagen mRNAs in VIM+/+ MEFs. Importantly, WF-A did not affect the half-life of either collagen mRNA (Figure 4D).

Since WF-A did not alter half-lives of collagen mRNAs in the absence of vimentin, we concluded that the effect of WF-A on stability of collagen mRNAs is dependent on its effect on vimentin filaments. This explains the greater effect of WF-A on collagen mRNA steady state level seen in VIM+/+ MEFs compared to VIM−/− MEFs.

### Withaferin-A Inhibits TGF-β1 Induced Type I Collagen Expression and COL1A2 Promoter Activity

The observation that WF-A still affected the expression of collagen mRNAs in VIM−/− MEFs, although to a lesser extent then in VIM+/+ MEFs, suggested that the mechanism by which WF-A decreases expression of collagen mRNAs is not solely limited to decreasing the half-lives. Because another mechanism could also contribute to the effect, we decided to investigate if WF-A also exerts its effect at the level of transcription. Transcription of collagen genes can be stimulated by TGF-β1 through the TGF-β/Smad signaling pathway. To assess if transcription induced by TGFβ is independent on vimentin filaments, which serve to stabilize collagen mRNAs, we treated VIM+/+ and VIM−/− MEFs with TGFβ (Figure S3). Although VIM−/− cells had a lower initial level of collagen mRNAs (compare lanes 2 and 4), TGFβ increased their expression two fold. A similar two fold increase was seen in VIM+/+ cells (compare lanes 1 and 3), suggesting that vimentin filaments are not needed for TGFβ stimulation. This prompted us to investigate if WF-A may have additional effects on the TGFβ signaling.

**Figure 5:**
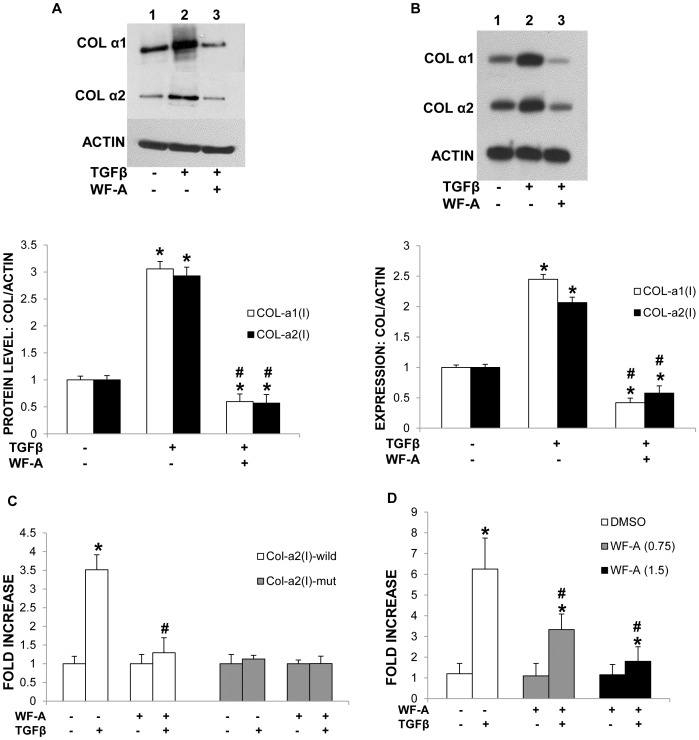
WF-A inhibits TGF-β1 induced expression of type I collagen. A. WF-A inhibits TGF-β1 induced increase in collagen polypeptides. HLFs were treated with DMSO (lanes 1 and 2) or 1.5 µM WF-A (lane 3) for 1 h and then treated with TGF-β1 (lanes 2 and 3) for 24 h. Expression of collagen α1(I) (COLα1) and collagen α2(I) (COLα2) polypeptides in cell lysates was measured by Western blot. Loading control: actin. Bottom panel: levels of collagen α1(I) and α2(I) polypeptides were normalized to that of actin and plotted from three independent experiments. The error bars represent ±1 SEM. B. WF-A inhibits induction of collagen mRNAs by TGF-β1. Experiment as in A, except collagen mRNAs were analyzed by RT-PCR. Loading control: actin. Bottom panel: levels of collagen α1(I) and α2(I) mRNAs were normalized to that of actin mRNA and plotted from three independent experiments. The error bars represent ±1 SEM. C. WF-A inhibits transcriptional activation of collagen α2(I) promoter. Luciferase reporter gene containing wild type collagen α2(I) promoter (open bars) or collagen α2(I) promoter with the mutated TGF-β1-responsive element (black bars) were transfected into HLF. After treatment with WF-A and TGF-β1, luciferase activity was normalized to the co-transfected internal control gene β-GAL. The plot is from three independent transfections performed in duplicate. Error bars represent ±1 SEM. D. WF-A inhibits transcriptional activation of a SMAD responsive promoter. Experiment as in C, except reporter gene containing (CAGA)_12_MLP promoter was used and WF-A was used at concentrations 0.75 µM (gray bars) and 1.5 µM (black bars). * represents p<0.05 and # represents p<0.01.

Stimulation of HLFs by TGF-β1 increased levels of collagen α1(I) and α2(I) polypeptides by 3.0 and 2.8 fold, respectively (Figure 5A). However, a TGF-β1 induced increase in expression of collagen α1(I) and α2(I) polypeptides was abolished in cells pretreated with 1.5 µM WF-A (Figure 5A, compare lanes 2 and 3). WF-A, not only abolished the induction of collagen by TGF-β1, but also reduced its level below that of control cells (lane 1). RT-PCR analysis revealed that TGF-β1 increased the steady state levels of collagen α1(I) and α2(I) mRNAs 2.5 and 2 fold, respectively (Figure 5B, lane 2). This induction was completely abolished in cells pretreated with 1.5 µM of WF-A (Figure 5B, lane 3). This treatment even reduced collagen mRNAs to the level lower than that of unstimulated cells.

TGF-β1 stimulates transcription of collagen α2(I) gene (COL1A2) by inducing binding of Smad-containing complex to the TGF-β1 responsive element (TbRE) located between nucleotides −313 and −250 of the promoter. To determine if WF-A can inhibit the TGF-β1 stimulated transcriptional activity of COL1A2 promoter, primary HLFs were transiently transfected with a –378-COL1A2/LUC reporter gene. This gene was driven by the wild type COL1A2 promoter containing 378 nt (Col-α2(wt)-Luc). A control gene had the mutated TbRE element in the same promoter (Col-α2(mut)-Luc). These reporter genes have been described before [25] and they contain only the promoter sequence and a short 5′ UTR, without 5′ stem-loop. 24 h after transfection, the cells were treated with WF-A (1.0 µM), followed by stimulation with TGF-β1 (5 ng/ml). The mutant Colα2(I) promoter failed to respond to TGF-β1, expression of LUC protein was the same in stimulated and unstimulated cells (Figure 5C, gray bars). However, TGF-β1 stimulated the transcriptional activity of the wild type COL1A2 promoter by 3.5 fold compared to unstimulated cells (Figure 5C, open bars). Treatment of cells with 1.0 µM of WF-A completely inhibited the stimulation of transcription driven by wild type COL1A2 promoter construct and had no effect on transcription from the mutant promoter.

To confirm the above result, we used another TGF-β1 responsive reporter construct, containing 12 repeats of the Smad-binding element CAGA ((CAGA)12MLP-Luc construct). TGF-β induced this promoter 6 fold in HLFs (Figure 5D, open bars), but the induction was inhibited by about 50% in cells treated with 0.75 µM of WF-A (gray bars) and by about 75% with 1.5 µM of WF-A (black bars). This result suggests that, in addition to disrupting vimentin filaments and destabilizing collagen mRNAs, WF-A can also interfere with TGF-β1 induced transcription of collagen genes.

### Withaferin-A Blocks Smad3 Phosphorylation

**Figure 6:**
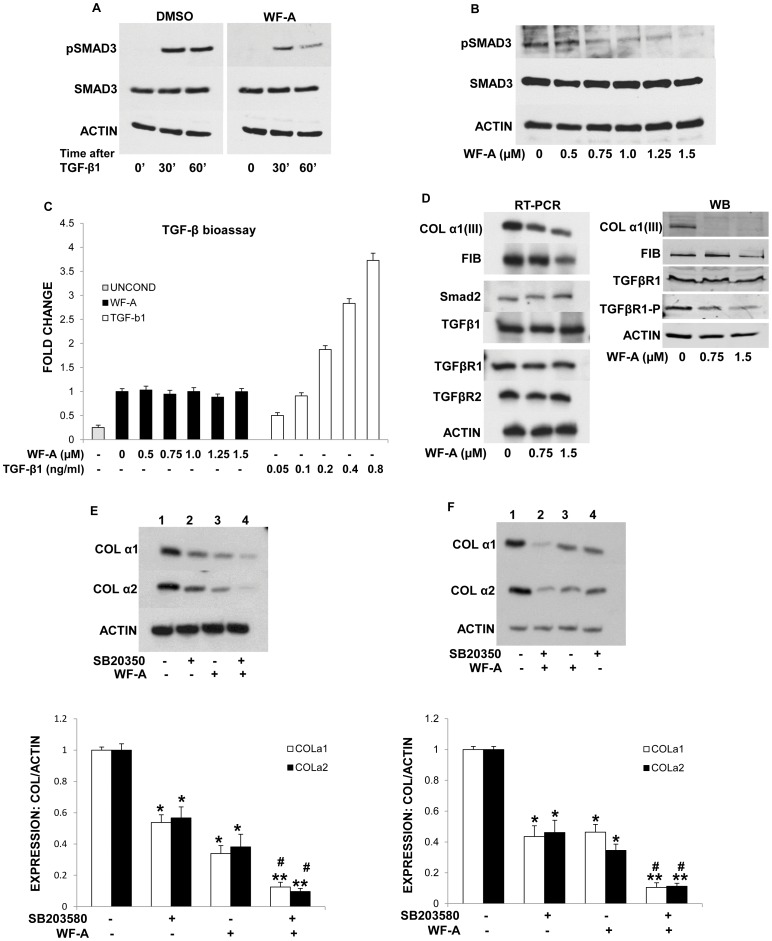
WF-A inhibits TGF-β1 signaling. A. WF-A inhibits TGF-β1 induced Smad3 phosphorylation. HLF were treated with DMSO (left panel) or WF-A (1.5 µM) (right panel) and stimulated by TGF-β1. Phospho-Smad3 and total Smad3 were measured by Western blot after indicated times. Loading control: actin. B. WF-A inhibits constitutive Smad3 phosphorylation in a dose dependent manner. Similar experiment as in A, HLF were treated with WF-A (0–1.5 µM). Loading control: actin. C. WF-A does not affect the amount of active TGF-β1 produced by HLF. HLF were treated with WF-A (0–1.5 µM) for 24 h. Conditioned medium from HLF was added to Ming lung epithelial cells (MLEC) stably transfected with TGF-β1 luciferase responsive promoter (PIA-Luc). Luciferase activity was measured in MLEC lysates. Gray bar: unconditioned medium, black bars: HLF conditioned medium, white bars: MLEC treated with recombinant TGF-β1 (0.05–0.8 ng/ml). Each bar represents three independent experiments analyzed in duplicate. Error bars: ±1SEM. D. Effect WF-A on expression of TGF-β1 relevant genes. mRNA of collagen III (COL α1(III)), fibronectin (FIB), Smad2, TGF-β1, TGF-β receptor 1 (TGFβR1) and TGF-β receptor 2 (TGFβR2) from control cells and HLF treated with WF-A (0.75–1.5 µM) were analyzed by RT-PCR (left panel). Protein COLα1(III), FIB, TGFβR1 and phospho-TGFβR1 were analyzed by Western blot (right panel). Loading control: actin. E. Synergistic effect of p38 MAPK inhibitor (SB203580) and WF-A. HLF were treated with SB203580 (10 µM, lane 2), WF-A (1.5 µM, lane 3) or both (lane 4), DMSO (lane 1). COLα1 and COLα2 mRNAs were analyzed after 24 h by RT-PCR. Loading control: actin mRNA. Bottom panel: expression of collagen α1(I) mRNA (open bars) and α2(I) mRNA (black bars) were normalized to actin mRNA and plotted from three independent experiments. The error bars: ±1SEM. F. Effect of SB203580 and WF-A in SCL. Similar experiment as in E. * represents p<0.05 and # represents p<0.01.

To gain an insight in to a step at which WF-A interrupts TGF-β signaling, we assessed the effect of WF-A treatment on the phosphorylation of Smad3. Phosphorylation of Smad3 was assessed by Western blot using phospho-Smad3 specific antibody that detects phosphorylation at serine residues S423/S425. Treatment of HLFs with 5 ng/ml TGF-β1 induced phosphorylation of Smad3 within 30 minutes after treatment and the phosphorylation persisted for 60 minutes (Figure 6A, left panel). WF-A pretreatment abrogated TGF-β1 induced Smad3 phosphorylation by about 50% at 30 and 60 minutes (Figure 6A, right panel). The level of total Smad3 was not altered by WF-A. The decreased Smad3 phosphorylation following treatment with WF-A suggests that WF-A interferes with activation of TGF-β1 dependent transcription factors. This was already suggested using the reporter gene with COL1A2 promoter (Figure 5C). In cultured fibroblasts, there is constitutive phosphorylation of Smad3, which can be further stimulated by exogenous TGF-β1 [33]. To determine if WF-A can reduce the level of constitutive p-Smad3 in cultured fibroblasts, we treated HLF with increasing concentrations of WF-A and analyzed pSmad3 by Western blot. WF-A reduced the level of p-Smad3 in cultured HLFs in dose dependent manner (Figure 6B). The total amount of Smad3 remained unaltered 24 h after treatment of HLFs with WF-A (Figure 6B). Taken together, WF-A inhibited both the basal, as well as TGF-β1 stimulated phosphorylation of Smad3, indicating that WF-A inhibits the TGF-β1 signaling pathway upstream of Smad3 phosphorylation. In addition to disruption of vimentin filaments, this may be an additional mechanism by which WF-A can reduce collagen expression.

It has previously been suggested that WF-A may decrease the release of cytokines from macrophages and other inflammatory cells [34]. It is, thus, possible that WF-A may interfere with synthesis, release and activation of latent-TGF-β from the cultured fibroblasts. In order to exclude this possibility, we compared the activity of TGF-β1 secreted from control and WF-A treated fibroblasts. To this end we utilized a TGF-β1 bioassay, where conditioned medium from fibroblasts is transferred to Ming lung epithelial cells (MLEC) stably expressing TGF-β1 inducible Luc reporter gene. If the conditioned medium contains active TGF-β1, an increase in luciferase expression must be observed [28]. As a positive control for the assay, we treated MLECs with increasing concentrations of recombinant TGF-β1. Adding exogenous TGF-β1 from 0.05 ng/ml to 0.5 ng/ml increased luciferase expression in dose dependent manner (Figure 6C, white bars). Then, we tested the effect of the conditioned medium of control cells and cells treated with WF-A in concentrations from 0 to 1.5 µM (black bars). The conditioned medium increased the reporter expression 2 fold compared to the medium that has not seen cells (gray bar) and this increase was comparable to the effect of 0.1 ng/ml of pure TGF-β1. There was no difference in the stimulatory effect of the conditioned medium whether the cells were treated with WF-A or not. This indicates that WF-A does not inhibit production, secretion or activity of TGF-β1 from cultured fibroblasts.

Another possible mechanism by which WF-A can inhibit TGF-β signaling pathway, is to down regulate the expression and/or activation of TGF-β receptors (TGF-βR1 and TGF-βR2) or Smad2. RT-PCR analysis showed that WF-A did not affect the levels of mRNAs encoding TGF-βR1, TGF-βR2 and Smad2 (Figure 6D, left panel). WF-A did not affect the mRNA level of TGF-β1, as already suggested by the experiment with the conditioned medium, but it decreased the expression of type III collagen and fibronectin, which are TGF-β responsive genes. This suggested that WF-A may interfere with the signaling from the TGF-β receptor and not with the expression of the receptor or Smad2.

Phosphorylation of TGF-βRI is critical for signal transduction upon TGF-β binding. Therefore, we measured the level of phosphorylated TGF-βRI upon treatment of HLF with WF-A. The total protein level of TGF-βR1 was unchanged, but the phosphorylation of TGF-βR1 decreased about 2 fold with 0.75 µM WF-A and about 4 fold with 1.5 µM WF-A (Figure 6D, right panel). These results indicate that WF-A blocks activation of TGF-βR1. This is consistent with previous results showing that WF-A reduced Smad3 phosphorylation, which is dependent on the active receptor (Figure 6B). Protein levels of two other TGF-β1 responsive genes, type III collagen and fibronectin were dramatically reduced. Together with the decrease in expression of type I collagen and decreased activity of TGF-β1 dependent promoters, this strongly suggests that WF-A treatment results in an inactive TGF-β receptor.

In addition to the canonical TGF-β/Smad pathway, TGF-β also can induce the activation of the p38 MAPK pathway mediated by the upstream TGF-β-activated kinase 1 (TAK1), a member of the MAPKKK family [35]. The TGF-β/p38 MAPK signaling pathway has also been implicated in TGF-β induced collagen expression. Based on our finding that WF-A inhibited phosphorylation of TGF-β receptor 1, we surmised that TGF-β/p38 MAPK pathway will also be affected. To test this, we treated HLF with 10 µM SB203580, a p38/MAPK inhibitor [24], alone, and in combination with WF-A. SB203580 alone decreased collagen α1(I) and α2(I) mRNAs two fold (Figure 6E), what was slightly less effect than WF-A alone (compare lanes 2 and 3). However, a combination of SB203580 and WF-A resulted in up to 10 fold reduction in the steady state levels of collagen α1(I) and α2(I) mRNAs (lane 4), suggesting a synergistic effect The experiments in SCL fibroblasts produced similar result (Figure 6F) and confirmed the potent additive effect of a combination of WF-A and p38 MAPK inhibitor on collagen expression. Taken together, these results show that WF-A inhibits TGF-β signaling starting from the receptor, what is further potentiated by the p38 MAPK inhibition.

### Withaferin-A Inhibits Activation of Hepatic Stellate Cells (HSC) and Decreases their Collagen I Production in vitro

**Figure 7:**
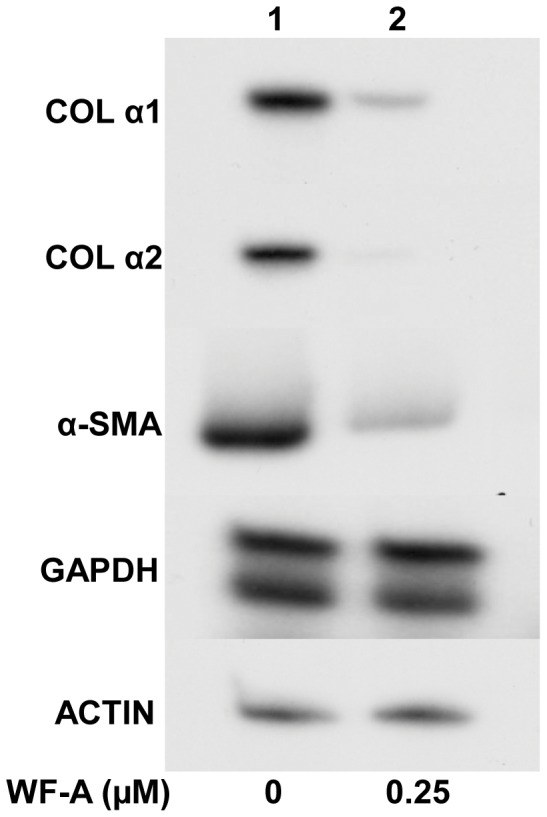
WF-A inhibits culture activation of primary rat HSC. HSC were isolated from rat livers and cultured for 2 days, when the cells were treated with DMSO (lane 1) or WF-A (0.25 µM, lane 2). At day 6, total RNA was analyzed for collagen α1(I) mRNA (COLα1), collagen α2(I) mRNA (COLα2) and α-smooth muscle actin mRNA (α-SMA) by RT-PCR. Loading controls: actin mRNA and GAPDH mRNA.

Activated HSC are the source of excessive production of ECM in chronic liver diseases and are, thus, the most relevant cell types for development of hepatic fibrosis. Following liver injury in vivo, HSC undergo activation, which is a transition from quiescent vitamin A-rich cells into proliferative, fibrogenic, and contractile myofibroblasts [7], [36]. Similar process can be reproduced in vitro by culturing HSC on plastic dishes, and is widely used as a model to study HSC activation. An activation of HSC is mediated by several cytokines, the most important being TGF-β1. Since our results demonstrated that WF-A can inhibit TGF-β1 signaling pathway, we further examined if WF-A can also inhibit the culture activation of quiescent hepatic stellate cells isolated from rat liver. After isolation, HSC were cultured for two days, then DMSO or sub-toxic does of WF-A (0.25 µM) were added to the medium. After incubation for additional 4 days, the cells were harvested and analyzed for expression of α-smooth muscle actin (α-SMA) mRNA, as a marker of activation. Figure 7 shows that WF-A at 0.25 µM significantly down regulated the expression of α-SMA in cultured primary HSC, suggesting that WF-A inhibited the culture activation of quiescent HSC in to myofibroblasts. WF-A treated HSC also expressed dramatically lower levels of collagen α1(I) and α2(I) mRNAs compared to the controls cells (Figure 7). There are many similarities in response to TGF-β between hepatic stellate cells and fibroblast in skin, lung and heart [37]. From the experiments with isolated rat HSC, as an in vitro model of fibroproliferative response driven by TGF-β1, we concluded that WF-A may have a potential to attenuate fibrosis *in vivo*.

### Withaferin-A Inhibits Isoproterenol-induced Myocardial Fibrosis in Mice

To validate if WF-A has an effect on collagen I expression, and development of fibrosis *in vivo*, we investigated if WF-A treatment can attenuate isoproterenol induced myocardial fibrosis. Isoproterenol is well known β-adrenergic agonist that has been used to induce experimental myocardial injury and fibrosis in mice and rats [38], [39], [40], [41]. Twelve weeks old (25–30 grams) 129 Svev male mice (n = 24) were randomly divided into four groups; Isoproterenol (ISO) group (n = 6), Isoproterenol and WF-A (ISO + WF-A) group (n = 6), WF-A group (n = 6) and vehicle (CONT) group (n = 6). Isoproterenol (50 mg/kg) was injected subcutaneously to the ISO and the ISO + WF-A groups for 2 consecutive days, whereas mice in the control groups received subcutaneous saline injections. WF-A (4 mg/kg) was injected intraperitoneally daily for 14 days into WF-A groups, whereas the vehicle was injected into the control group. 24 h after the last injection, hearts were removed, weighed, fixed and processed for histopathologic analysis. In addition, a heart tissue was snap frozen for analysis of protein and RNA.

**Figure 8:**
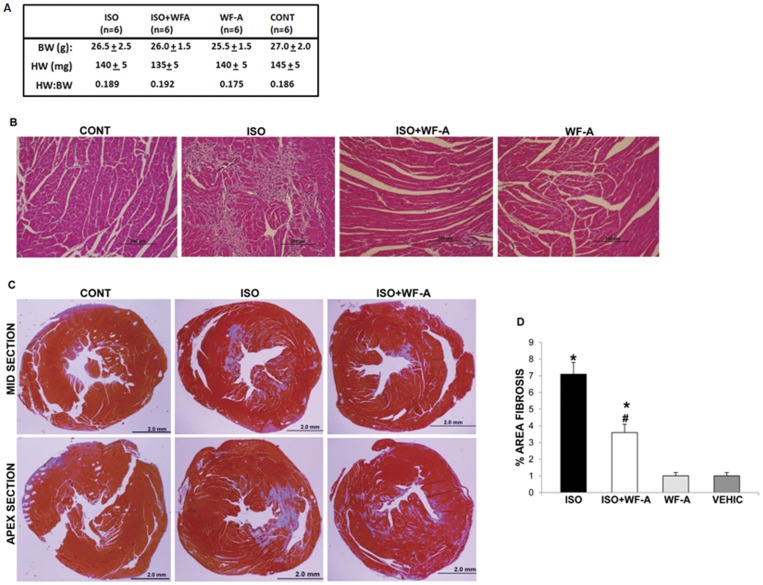
WF-A inhibits isoproterenol induced myocardial fibrosis in mice. A. Mice were injected with isoproterenol (ISO), isoproterenol + WF-A (ISO+WF-A), WF-A alone or vehicle (CON). Heart weight (HW) to body weight (BW) ratio (HW:BW) is shown as the mean ±1 SEM. B. WF-A protects against necrosis of heart tissue. H&E staining of a representative section of a heart from the control group (CONT), isoproterenol group (ISO), isoproterenol + WF-A group (ISO + WF-A) and WF-A group (WF-A). Scale bar: 200 µm. C. WF-A decreases isoproterenol induced myocardial fibrosis in mice. Masson’s trichrome staining of the base of the ventricle sections of hearts of control mice and mice treated with ISO and with ISO+WF-A. Two representative sections of each heart are shown with collagen staining in blue. Scale bar: 2 mm. D. Morphometric analysis of the percentage of fibrotic area of the hearts. Fibrosis areas within the sections (blue) were quantified using Image J software. The percentage of fibrosis was estimated from the ventricular and apex sections of each heart. Data represent ±1 SEM of six mice per group. * represents p<0.05 and # represents p<0.01.

An administration of Isoproterenol and/or WF-A didn’t change the weight of the heart, as the heart weight to body weight ratio (HW/BW ratio) didn’t significantly differ among the groups (Figure 8A), This suggested that Isoproterenol did not cause heart hypertrophy. However, two injections of Isoproterenol, resulted in significant necrosis of cardiomyocytes, which was still evident 12 days after its administration (Figure 8B, compare ISO panel to CONT panel). Histology of the hearts of WF-A treated animals (WF-A panel) was indistinguishable from the control animals, suggesting that WF-A has no toxicity to the heart. However, when WF-A was administered to Isoproterenol treated animals (ISO + WF-A panel) the heart necrosis was greatly reduced compared to animals treated with Isoproterenol alone (ISO panel). This suggests that WF-A reduced the initial isoproterenol injury and/or improved the recovery from it. To estimate the effect of WF-A on Isoproterenol induced heart fibrosis, Masson’s trichrome staining, which specifically stains collagen fibers blue, was done on two representative heart sections. One was from the middle of the heart around the level of papillary muscles and the other was from the apex of the heart (Figure 8C). The percentage of fibrotic area of each section was estimated from the extent of the Masson’s trichrome staining by one of the authors (J. Blackmon), blinded as to the groups. The percentage of fibrosis was also analyzed by Image-J software. Treatment of mice with WF- significantly decreased the development fibrosis (Figure 8C, compare the (ISO) and (ISO+WF-A) sections). The percent of area of fibrosis estimated from 6 animals was ∼7% in ISO alone group, compared to ∼3.5% in the ISO+WF-A groups (p<0.001) (Figure 8D). Thus, in addition to reducing necrosis (Figure 8B), WF-A also reduced Isoproterenol induced cardiac fibrosis by 50%.

**Figure 9:**
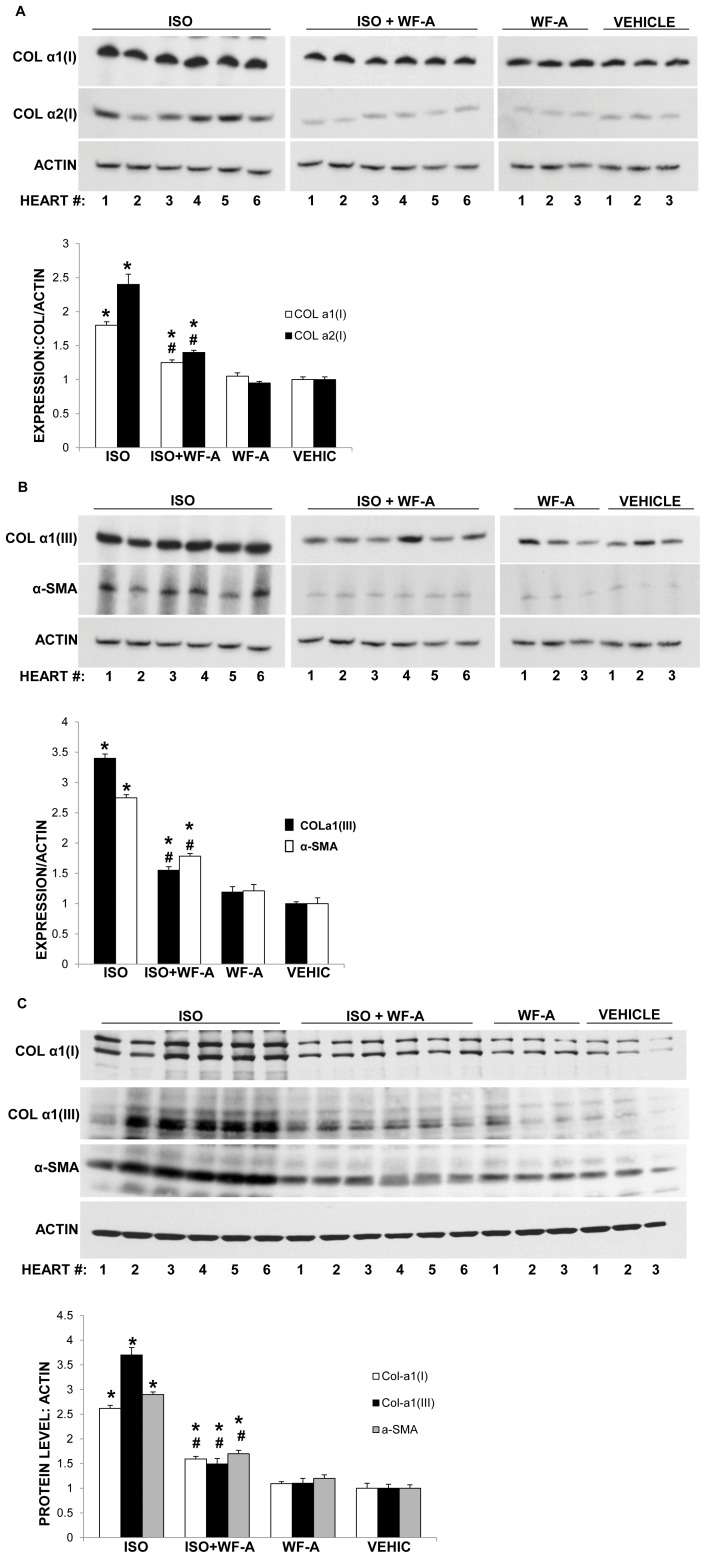
WF-A inhibits expression of α-SMA, type I, and type III collagens in isoproterenol induced cardiac fibrosis. A. WF-A decreased expression of collagen α1(I) and α2(I) mRNAs in experimental cardiac fibrosis. Total RNA was extracted from the heart tissue of the study groups and analyzed by RT-PCR for expression of collagen α1(I) mRNA (COLα1), collagen α2(I) mRNA (COLα2) and α-smooth muscle actin mRNA (α-SMA). Loading control: actin mRNA. The numbers indicate individual animals in each experimental group. Bottom panel: Expression of collagen α1(I) mRNA (open bars) and collagen α2(I) mRNA (black bars) was normalized to actin mRNA and plotted for each group. Error bars represent ±1SEM. B. WF-A inhibits expression of collagen α1(III) and α-smooth muscle actin mRNAs in isoproterenol induced cardiac fibrosis. Experiment as in A, except collagen α1(III) mRNA (COLIII) and α-smooth muscle actin mRNAs (α-SMA) mRNA were analyzed. C. WF-A reduces collagen α1(I) and α1(III) polypeptides and α-smooth muscle actin protein. Total protein extracted from the hearts of the study groups was analyzed for collagen α1(I) polypeptide, collagen α1(III) polypeptide and α-SMA protein by Western blot. Loading control: actin. The numbers represent individual animals in the experimental groups. Bottom panel: protein expression was normalized to actin expression and plotted for the groups. Collagen α1(I) polypeptide; open bars, collagen α1(III) polypeptide; black bars and α-SMA protein; gray bars. Error bars: ±1SEM. * represents p<0.05 and # represents p<0.01.

To further corroborate the effect of WF-A on cardiac fibrosis, we analyzed expression of type I and type III collagen, and α-SMA from heart tissue extracts by Western blot and RT-PCR. Collagen α1(I), collagen α2(I), and collagen α1(III) mRNAs were reduced 2 fold in the ISO+WF-A group compared with the ISO group (Figure 9A). α-SMA mRNA was also decreased in WF-A+ISO treated animals compared to ISO-treated animals and was similar to that of control animals (Figure 9B). Western blot analysis for type I and type III collagen and αSMA showed similar results as that obtained from the mRNA analysis (Figure 9C). Collagen I and III and αSMA polypeptides were decreased by ∼50% in ISO+WF-A group compared with the ISO group. This is in full agreement with the reduced collagen staining in the histology slides (Figure 8C).

The use of an animal model of cardiac fibrosis clearly showed that WF-A can attenuate fibrosis, improve tissue damage and suppress collagen expression *in vivo.* Decreased expression of αSMA in a heart tissue suggested that WF-A prevented activation of cardiac fibroblasts into myofibroblasts *in vivo*, what is in agreement with the inhibition of activation of HSC *in vitro* (Figure 7). All together these results indicate a significant anti-fibrotic effect of WF-A on fibrosis *in vivo*, including but not limited to cardiac fibrosis.

## Discussion

Increasing understanding of the key factors involved in the regulation of normal and excessive collagen expression is essential for discovery of potential targets of anti-fibrotic drugs. However, a discovery of anti-fibrotic interventions hasn’t kept pace with the progresses made in the understanding of molecular mechanisms, that mediate the initiation, and progression of fibrosis [42]. Currently, no anti-fibrotic agents are licensed for use in humans [9], [42], [43], [44], [45], [46].

In fibroproliferative disorders, a tissue injury is associated with activation of collagen producing cells, followed by the excessive synthesis and deposition of type I collagen [5]. In activated cells, the increased expression of collagen is the result of the increased rate of transcription of collagen I genes and the increased half-life of collagen I mRNAs [47]. Targeting molecules involved in transcriptional and posttranscriptional regulation of expression of type I collagen could lead to potential anti-fibrotic therapies [46].

We recently reported that vimentin filaments bind and stabilize type I collagen mRNAs [16]. WF-A, a bioactive substance obtained from a widely used Indian herbal plant *Withania sominifera*, was reported to disrupt vimentin intermediate filaments in endothelial cells, astrocytes and some vimentin containing cancer cell lines [18], [30], [31]. Based on our recent finding that the integrity of vimentin filaments is needed for stability of type I collagen mRNAs, we hypothesized that WF-A treatment may reduce collagen expression by destabilizing collagen mRNAs. No prior study has examined the effects of WF-A on collagen synthesis *in vitro* or in animal models of fibrosis.

Using fibroblasts from different tissues (lung, skin, heart) and HSC from liver, we demonstrated that WF-A can disrupt vimentin filaments and inhibit collagen expression by >50%. This was achieved at sub-toxic range (0.25–1.5 µM) of WF-A concentrations, the dose depending on the type of fibroblasts. WF-A reduced the production of collagen I from fibroblasts through at least two mechanisms.

First, WF-A caused a decrease in stability of collagen I mRNAs. It is almost certain that this effect is due to the ability of WF-A to disrupt vimentin filaments (Figure 1), because WF-A induced destabilization of collagen mRNAs was seen only in fibroblasts containing vimentin (Figures 3 and 4) and not in vimentin knock-out cells (Figure 4). This result is in line with our recently published findings that lack of vimentin or disruption of vimentin using dominant-negative desmin destabilizes type I collagen mRNAs [16]. The destabilizing effect WF-A on collagen mRNAs is, therefore, consistent with the role of vimentin in posttranscriptional regulation of collagen expression.

Second, WF-A inhibited TGF-β1 induced transcriptional activation of TGF-β1-responsive promoters, including that of COL1A2 (Figure 5C and 5D). WF-A also inhibited expression of TGF-β1 responsive genes, type III collagen and fibronectin (Figure 6D). TGF-β1 is the single most potent profibrogenic factor involved in the initiation and maintenance of fibrogenesis [36], [37], [48], [49], [50], [51]. Approaches aimed at interfering with the TGF-β1 signaling pathway are being studied as a potential cure for fibrosis [42], [44], [45]. *In vivo,* experiments using different strategies to block TGF-β1 have demonstrated significant anti-fibrotic effects in several organ systems including the liver, lung and heart [52], [53], [54]. Using luciferase reporter assays for assessing transcriptional activity of collagen α2(I) promoter, we demonstrated that WF-A reduced the TGF-β1 induced transcription.

In addition, WF-A interrupted the TGF-β1 signaling pathway, as demonstrated by the markedly decreased phosphorylation of Smad3 (Figure 6A). Moreover, even in unstimulated fibroblasts, WF-A abolished the level of constitutive phosphorylation of Smad3 in a dose dependent manner (Figure 6B). This suggested that WF-A must act at the level upstream of Smad3 phosphorylation, either by inhibiting TGF-β receptor signaling or activity of TGF-β itself.

WF-A did not significantly change the level of TGF-β1 mRNA or the amount or activity of TGF-β secreted from fibroblasts (Figure 6C and 6D). This made the possibility that WF-A acts on TGF-β1 unlikely. The mRNA levels of TGF-βR1 and TGF-βR2 and Smad2 were also unchanged. While the total protein level of TGFβ1-R1 was unaffected by WF-A, the phosphorylated form of TGFβ1-R1 was drastically reduced. This suggests that WF-A inhibited activation of the receptor by preventing phosphorylation of the signal transducing subunit 1. The exact mechanism how WF-A suppresses activation of TGF-β receptor is not clear. It may bind directly to one of the subunits of the receptor making it nonfunctional or it may prevent TGF-β docking to the receptor. Lower activity of the receptor would result in a decrease of Smad3 phosphorylation, as well as in lower activity of the alternative, Smad-independent, p38 MAPK pathway. This is precisely what we have observed. WF-A seems to potentiate the effect of p38 MAPK inhibitor (SB203580) on TGF-β/p38 MAPK pathway, because the combination of WF-A and SB203580 resulted in synergistic effect on collagen expression (Figure 6E and 6F). This suggests that combination of WF-A and SB203580 may be a more potent anti-fibrotic therapy.

The effect of WF-A on TGF-β1 signaling was unrecognized so far, and we are the first to report it. So far it was reported that WF-A can bind to a protein in NF-kB signaling pathway and inhibit activation of NF-kB and subsequent pro-inflammatory response [55]. WF-A–protein complex was stable and its formation could be attributed to the anolide structure of WF-A.

Activation of quiescent HSC into myofibroblasts can be achieved by culturing freshly isolated HSC *in vitro*, this mimics the profibrotic activation of these cells *in vivo*. Such process is not possible with cardiac or other fibroblasts, therefore, we used HSC as a model of *in vitro* fibrogenesis. WF-A treatment abrogated culture activation of quiescent HSC into activated HSC, as indicated by the reduced expression of the marker of activation, α-smooth muscle actin (α-SMA, Figure 7). Since TGF-β has a critical role in activation of quiescent HSC [37], the delay in activation of HSC suggested that this effect of WF-A may be due to inhibition the TGF-β signaling pathway and that WF-A may be effective against fibrosis *in vivo*.

Novel drugs and approaches to slow the progression of fibrosis in organs like heart, liver, lungs and pancreas are highly needed [56]. For example, myocardial fibrosis, an excessive accumulation of collagen fibers within the cardiac interstitium, occurs in several cardiac diseases: hypertensive heart disease, myocardial infarction, hyperterophic cardiomyopathy, idiopathic intersitial cardiac fibrosis, and decompensated congestive heart failure of any etiology [57], [58], [59], [60]. In HDD, type I and type III collagen content of the myocardium is increased 2 fold as a consequence of a number of hemodynamic, neuro-hormonal and cellular factors [61]. The presence of fibrosis is associated with deterioration of cardiac function and transition to heart failure in these patients [3], [62] [63], [64].

In recognition of the importance of treating fibrosis in heart diseases, we studied the effect of WF-A administration on the development of isoproterenol induced myocardial fibrosis. Isoproterenol is a well-known β-adrenergic agonist that produces positive inotropic and chrontropic effects. There are several established animals models of cardiac fibrosis induced by isoproterenol [38], [39]. We used a dose of isoproterenol that induces moderate cardiac fibrosis, as seen in hypertensive patients [62]. WF-A treatment resulted in a 50% reduction in the degree of myocardial fibrosis as assessed by evaluation of Masson’s trichrome stained heart sections. Fibrosis was reduced in the upper sections of the ventricles as well as in the apex sections (Figure 8B, 8C, and 8D). Consistent with the histologic findings, analysis of collagen proteins and mRNAs in the heart tissue revealed 50% decreased expression of collagen α1(I), α2(I), and α1(III) mRNA and protein (Figure 9A). In addition, there was a significant reduction in the level of α-SMA expression (Figure 9B) suggesting that WF-A prevented activation of fibroblasts into myofibroblasts in the hearts, similar to what was observed in HSC. Significantly, the degree of cardiac necrosis persisting 12 days after the last isoproterenol injection was much lower if WF-A had been administered daily (Figure 8B). This suggests that WF-A may have a cardio protective activity, as well as anti-fibrotic activity. Such dual effects are desirable, as exampled by two most successful anti-hypertensive drugs, Lisinopril (an ACE inhibitor) and Losartan (an Aldosterone receptor blocker). These drugs improve heart function by lowering blood pressure and by inhibiting collagen I synthesis [65].

The benefits of using WF-A for therapy include history of safe use in traditional medicine and anti-inflammatory properties [66], that may prove even more beneficial in fibrotic diseases with underlying chronic inflammation [67]. In line with this, we did not observe any adverse effects of WF-A on heart histology; this is consistent with other *in vivo* studies using doses as high as 30 mg/kg [23], [68], [69]. Our results revealed the two steps at which WF-A can act to inhibit fibrogenesis; inhibition of TGF-β1 signaling with reduced transcription of collagen genes and interruption of vimentin network with destabilization of collagen mRNAs.

In summary, our studies reveal for the first time the anti-fibrotic activities of WF-A *in vitro* and *in vivo* and its mechanisms of action. These findings provide a strong base for the further exploration of WF-A as a therapeutic drug against fibroproliferative diseases, including but not limited to cardiac interstitial fibrosis. Since activation of HSC is central for development of liver fibrosis, and WF-A is a potent inhibitor of HSC activation, the logic next step is to evaluate the anti-fibrotic effect of WF-A in mouse models of liver fibrosis.

## Supporting Information

Figure S1 **Disruption of vimentin filaments in human fibroblasts changes the shape of cells.** Primary human lung fibroblasts (A) and scleroderma fibroblasts (B) were treated for 2 h with DMSO (left panels) or with 1.0 µM of WF-A (right panels) and fixed on a glass cover slip. Nuclear staining was done by DAPI and cell shape was imaged by differential interference contrast (DIC) mode with 63x phase contrast planapochromat oil objective. The images represent a reconstructed two-dimensional view.
(TIF)

Figure S2 **Viability of fibroblasts after WF-A treatment.** A. Viability of primary human lung fibroblasts (HLF) after 24 h of treatment with the indicated concentrations of WF-A. Apoptosis was determined with caspase glo 3/7 assay kit and the relative luminescence is shown as the percentage of that of control cells. B. Viability of scleroderma fibroblasts (SCL). C. Viability of rat cardiac fibroblasts (RCF) D. Viability of primary rat hepatic stellate cells (HSC). E. Viability of wild type mouse embryonic fibroblasts (VIM+/+MEFs) and vimentin knock-out mouse embryonic fibroblasts (VIM−/−MEFs). Error bars represent ±1SEM, determined from three independent experiments.
(TIF)

Figure S3 **TGF-β1 stimulation of VIM+/+ and VIM−/− fibroblasts increases the level of collagen α1(I) and α2(I) mRNAs.** Wild type (Vim+/+) and vimentin knock out (VIM−/−) mouse embryonic fibroblasts were stimulated with 5 ng/ml TGF-β1 (lanes 1 and 3) for 24 h. Level of collagen α1(I) and α2(I) mRNAs was measured by RT-PCR. Collagen mRNA levels from unstimulated cells is shown in lane 2 and 4. Loading control: GAPDH.
(TIF)
