# Supplementary figures and images for: Withaferin-A Reduces Type I Collagen Expression In Vitro and Inhibits Development of Myocardial Fibrosis In Vivo
Source: PLoS One. 2012 Aug 10;7(8):e42989. doi: 10.1371/journal.pone.0042989 (PMC3416765; doi:10.1371/journal.pone.0042989)

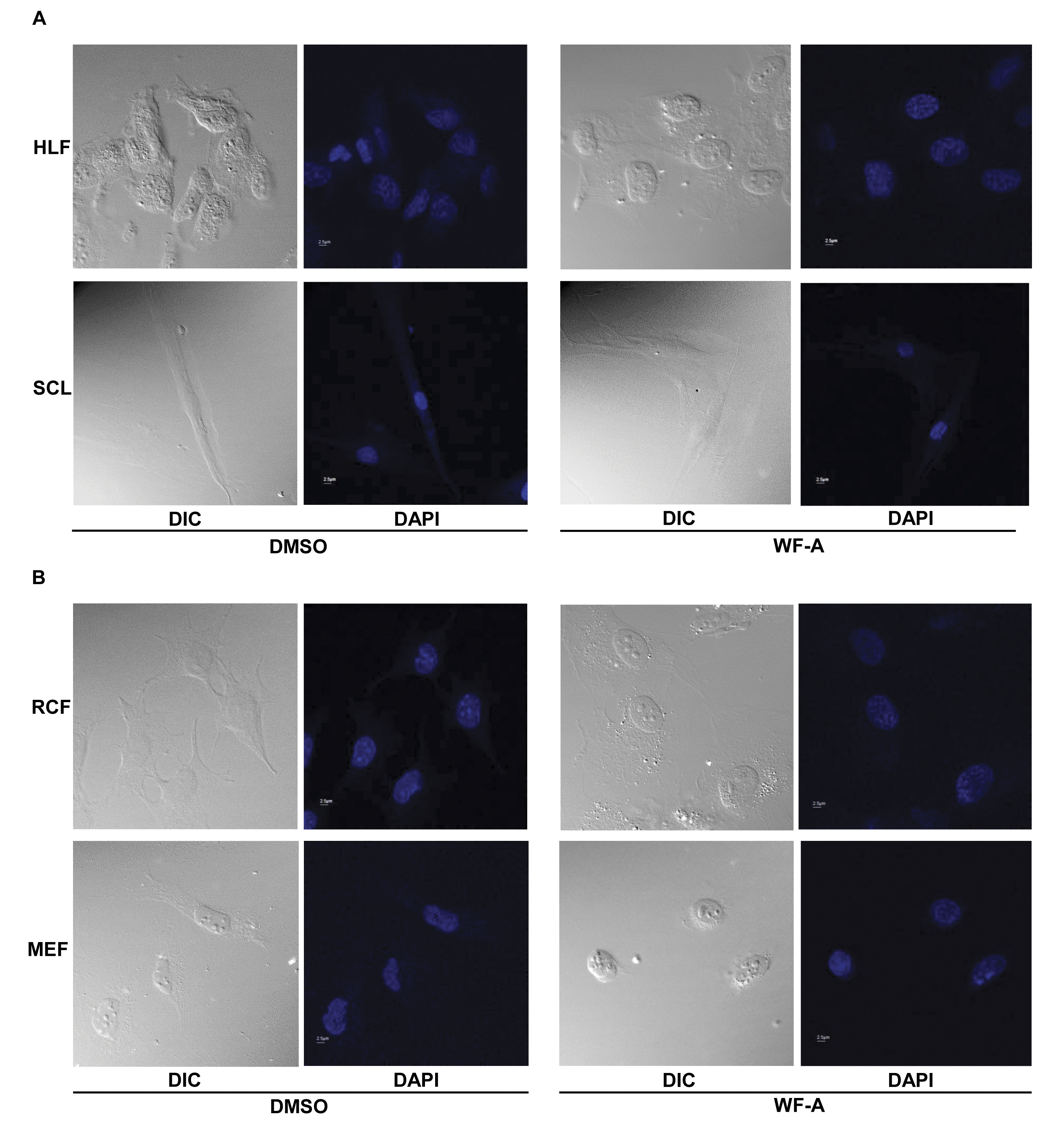

Supplement: Figure S1 — Disruption of vimentin filaments in human fibroblasts changes the shape of cells. Primary human lung fibroblasts (A) and scleroderma fibroblasts (B) were treated for 2 h with DMSO (left panels) or with 1.0 µM of WF-A (right panels) and fixed on a glass cover slip. Nuclear staining was done by DAPI and cell shape was imaged by differential interference contrast (DIC) mode with 63x phase contrast planapochromat oil objective. The images represent a reconstructed two-dimensional view. (TIF) [file pone.0042989.s001.tif]

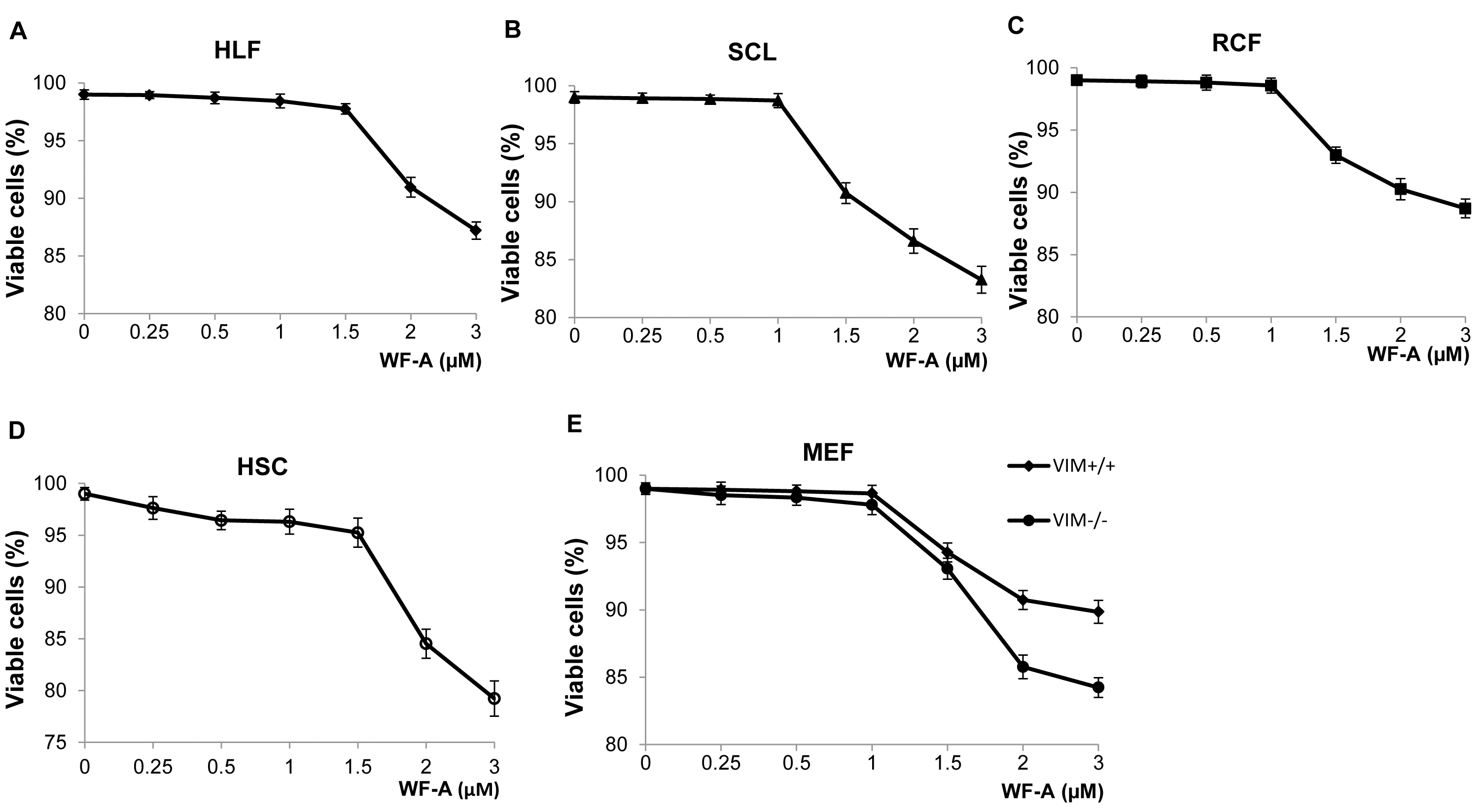

Supplement: Figure S2 — Viability of fibroblasts after WF-A treatment. A. Viability of primary human lung fibroblasts (HLF) after 24 h of treatment with the indicated concentrations of WF-A. Apoptosis was determined with caspase glo 3/7 assay kit and the relative luminescence is shown as the percentage of that of control cells. B. Viability of scleroderma fibroblasts (SCL). C. Viability of rat cardiac fibroblasts (RCF) D. Viability of primary rat hepatic stellate cells (HSC). E. Viability of wild type mouse embryonic fibroblasts (VIM+/+MEFs) and vimentin knock-out mouse embryonic fibroblasts (VIM−/−MEFs). Error bars represent ±1SEM, determined from three independent experiments. (TIF) [file pone.0042989.s002.tif]

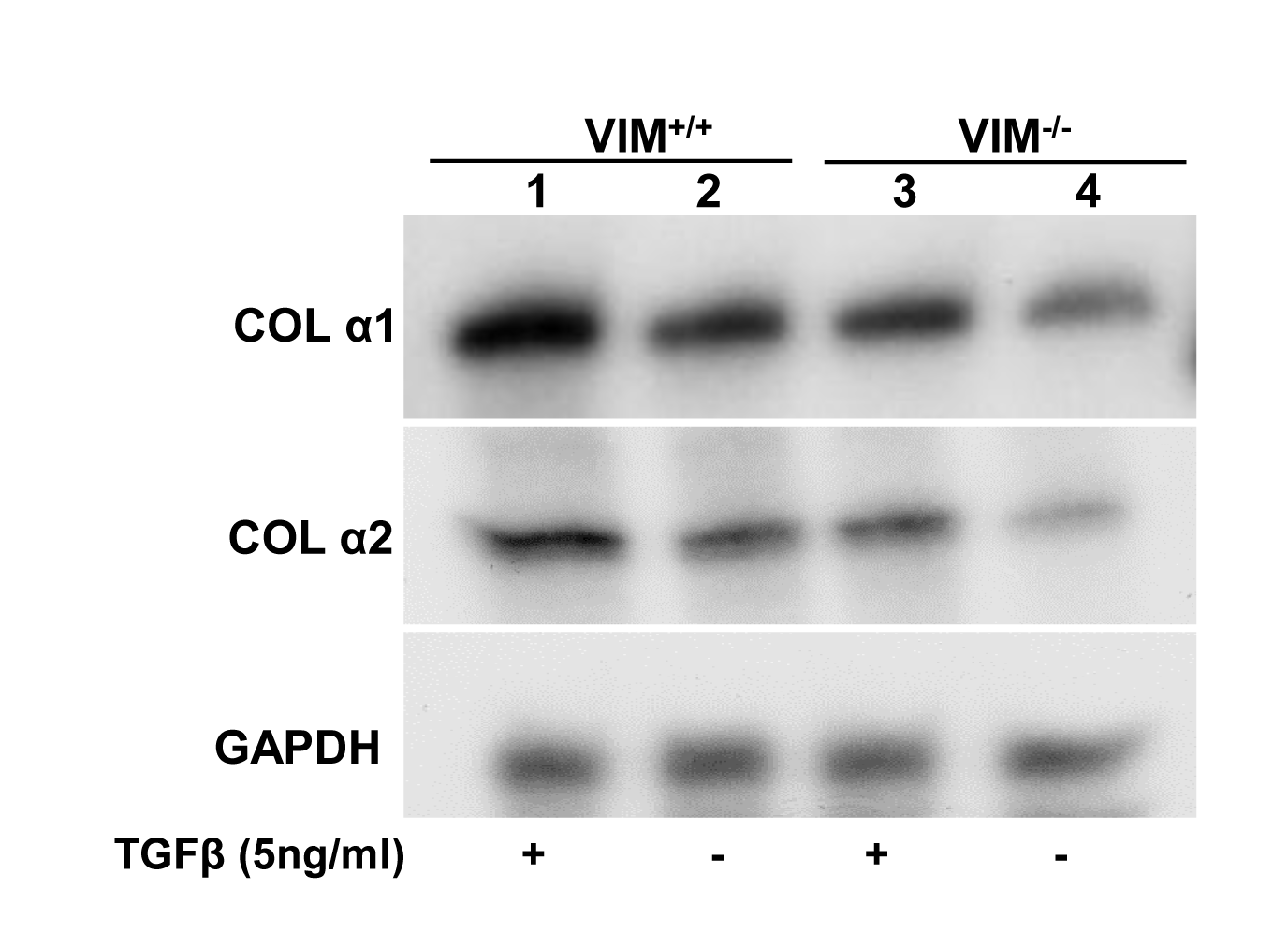

Supplement: Figure S3 — TGF-β1 stimulation of VIM+/+ and VIM−/− fibroblasts increases the level of collagen α1(I) and α2(I) mRNAs. Wild type (Vim+/+) and vimentin knock out (VIM−/−) mouse embryonic fibroblasts were stimulated with 5 ng/ml TGF-β1 (lanes 1 and 3) for 24 h. Level of collagen α1(I) and α2(I) mRNAs was measured by RT-PCR. Collagen mRNA levels from unstimulated cells is shown in lane 2 and 4. Loading control: GAPDH. (TIF) [file pone.0042989.s003.tif]
